# Bioactive compounds by microalgae and potentials for the management of some human disease conditions

**DOI:** 10.3934/microbiol.2023004

**Published:** 2023-02-07

**Authors:** Chijioke Nwoye Eze, Chukwu Kenechi Onyejiaka, Stella Amarachi Ihim, Thecla Okeahunwa Ayoka, Chiugo Claret Aduba, Johnson k. Ndukwe, Ogueri Nwaiwu, Helen Onyeaka

**Affiliations:** 1 Department of Science Laboratory Technology, University of Nigeria Nsukka; 2 Department of Microbiology, University of Nigeria Nsukka; 3 Department of Pharmacology and Toxicology, Faculty of Pharmaceutical, University of Nigeria Nsukka; 4 Department of Biochemistry, University of Nigeria Nsukka; 5 UNESCO International Centre for Biotechnology, University of Nigeria Nsukka; 6 School of Chemical Engineering, University of Birmingham, Edgbaston, Birmingham, B15 2TT, United Kingdom

**Keywords:** active compounds, carotenoids, Alzheimer's disease, oxidative stress

## Abstract

Microalgae biomasses are excellent sources of diverse bioactive compounds such as lipids, polysaccharides, carotenoids, vitamins, phenolics and phycobiliproteins. Large-scale production of these bioactive substances would require microalgae cultivation either in open-culture systems or closed-culture systems. Some of these bioactive compounds (such as polysaccharides, phycobiliproteins and lipids) are produced during their active growth phase. They appear to have antibacterial, antifungal, antiviral, antioxidative, anticancer, neuroprotective and chemo-preventive activities. These properties confer on microalgae the potential for use in the treatment and/or management of several neurologic and cell dysfunction-related disease conditions, including Alzheimer's disease (AD), AIDS and COVID-19, as shown in this review. Although several health benefits have been highlighted, there appears to be a consensus in the literature that the field of microalgae is still fledgling, and more research needs to be carried out to ascertain the mechanisms of action that underpin the effectiveness of microalgal compounds. In this review, two biosynthetic pathways were modeled to help elucidate the mode of action of the bioactive compounds from microalgae and their products. These are carotenoid and phycobilin proteins biosynthetic pathways. The education of the public on the importance of microalgae backed with empirical scientific evidence will go a long way to ensure that the benefits from research investigations are quickly rolled out. The potential application of these microalgae to some human disease conditions was highlighted.

## 1. Introduction

Microalgae are considered a healthy food due to their balanced nutritional and bioactive components. The diversity of microalgae species also gives rise to various nutritional and bioactive molecules, which makes microalgae the richest natural resource for nutritional and bioactive components. In addition, the absence of cellulose cell wall makes for easy digestion of biomass after consumption. Interestingly, studies have shown that nutritional supplements, such as vitamin C, vitamin E and omega-3 fatty acids, which are contained in healthy foods [1],[2] and found in microalgae, can reduce the risk of some health disorders [3],[4]. This has heightened the growing interest in the search for a nutraceutical as a possible replacement for synthetic drugs which have a myriad of side effects for the treatment or prevention of many diseases.

The production of microalgae biomass can be cost-effective, as basic resources, such as water, carbon(IV) oxide and sunlight, required for cultivation are cheap and readily available. Many species, such as *Chlorella*, *Dunaliella* and *Haemaotococcus*, have been commercialized as health food due to their ability to accumulate some nutritional and bioactive compounds [5]. The multiple bioactive compounds accumulated in microalgae biomass, such as vitamins C and E, lipids and carotenoids [6], phycobiliproteins and polysaccharides, confer on it a wide range of possible medical applications, amongst which is the treatment of Alzheimer's disease [7], HIV and SARS-CoV-2 [8]. Microalgal-derived antioxidants, according to Ataie et al. [9], prevent radical-induced neuronal damage and AD progression by scavenging free radicals which harm the brain cells. Microalgae accumulate these antioxidants as a means of protecting themselves under stressed conditions. These stressed conditions have been artificially created by many researchers to induce the accumulation of antioxidants in microalgae by metabolic or environmental engineering [6]. However, currently, genetic engineering is used to manipulate microalgae for the high accumulation of these antioxidants / bioactive molecules. Each of the manipulative strategies for high induction of bioactive molecules affects the cellular metabolism due to adjustments of some metabolic pathways that lead to the production of such desired bioactive molecules. Hence, there is a need to understand the biosynthetic pathway for the production of these bioactive molecules to enable researchers to identify easily the points of manipulation for the induction of bioactive molecules. Although there were reviews in the literature that addressed the bioactive compounds by microalgae [10],[11], the provision of various biosynthetic pathways for the various bioactive molecules by microalgae as discussed in this review has not been carried out. This review focused on bioactive molecules by microalgae, microalgae biomass production, bioactive compounds biosynthetic pathways and mechanisms of action, which form the basis of curative/preventive potentials of microalgae against several diseases.

The work was carried out using the standard narrative review method. To generate phrases for the search activities, “benefits of microalgae” and “microalgae uses” were used for a search on the databases Web of Science, PubMed, Google Scholar and Scopus. Duplicate reports generated were left out, after which the carotenoids and phycobilin proteins biosynthetic model pathways were developed.

## 2. Microalgae biomass

Microalgae are known to be very diverse photosynthetic unicellular eukaryotic or prokaryotic organisms, believed to be among the earlier forms of life [5],[12]. They were first discovered as a source of food because of their rich nutritive value, and later, some species were commercially produced as healthy foods in some parts of Asia [5],[13]. The health-related nature of microalgae is predicated on some intracellularly-produced bioactive molecules which can either be extracted from the cell biomass or used as whole cell biomass depending on the end product. The accumulation of these bioactive molecules in microalgae, which is species-specific [14] and under varying culture conditions depending on the bioactive molecule of interest, has endeared microalgae biomass to pharmaceutical, food, chemical, textile and cosmetic industries. Due to the micro size of the microalgae species, biomass production is indispensable for commercial applications either as bioactive molecule extracts or as the whole cell.

Various types and designs of photobioreactors have been developed and reviewed elsewhere [15],[16], although new designs for cost-efficient production of microalgae biomass as well as for enhanced specific or simultaneous accumulation of many bioactive molecules are still emerging [6],[17]. However, the closed system is recommended for products meant for human consumption due to the high level of sterility ensured during production [15],[16]. Furthermore, the closed system can be optimized for high productivity to leverage the high cost of photobioreactors. Microalgae biomass cultivation requires potable or non-potable water, light (natural or artificial) and carbon sources (organic or inorganic), carbon(IV) oxide (from air or exhaust fumes of machines) [17]. However, the cultivation conditions can be different depending on the resources utilized during cultivation. For instance, the phototropic condition entails cultivation using light and inorganic carbon sources, while the mixotrophic or heterotrophic condition entails cultivation using organic carbon sources and light or without light, respectively [17]. Each of the cultivation conditions can be employed during microalgae biomass production using cost-efficient and contamination-prone open culture systems (ponds, lakes, etc.) or cost-intensive and sterile-conditioned closed culture systems (photobioreactors) [6]. Although the open culture system is cheap due to ease of design and readily available resources, it is limited by culture contamination, a requirement for space and a lack of culture control. For instance, Eze et al. [17] reported an airlift photobioreactor inclined with a reflective broth guide for enhanced light utilization by microalgae cells during cultivation. Several strategies have been employed for improved biomass productivity by microalgae which range from metabolic (media components) to genetic (genes and nucleic acids), environmental (temperature, salt concentration, pH, etc.) and culture system (one stage, two stages) engineering [18].

Each of these engineering strategies influences the diverse biosynthetic pathway leading to the biomass accumulation of various bioactive molecules. For example, when subjected to high light conditions, most carotenoids and chlorophyll contents of the marine microalga *Chlamydomonas* sp. JSC4 decreased, while zeaxanthin and antheraxanthin contents increased [19]. Another study [20] on the effect of light found that there was good mixotrophic growth and savings of energy for glucose in a continuous light-deficient condition. To reduce costs that make large-scale cultivation expensive, a mathematical model using the cultivation of *Tetradesmus obliquus* has been developed [21]. In the model, it was suggested that biomolecule production prediction in a particular condition could improve time and productivity and make the process more profitable.

Using western blotting, it was found by Guo et al. [22] that overweight protection was achieved by microalgae polysaccharides through increased lipolysis and decreased lipogenesis in the liver. Other investigators [23] found that the metabolic rewiring in the tricarboxylic acid (TCA) intermediates, amino acid metabolism and starch metabolism helps the cells to concentrate carbon pools for producing neutral lipids. It has also been suggested [24] that simultaneous inorganic carbon supply and pH control can be a cost-effective measure without compromising biomass and lipid productivities if the sole use of bicarbonates is applied in microalgae culture.

In another study, using semi-continuous cultivation with a medium replacement ratio of 75% resulted in a higher lutein productivity and lutein concentration of 6.24 mg/L/d and 50.6 mg/L, respectively, which were markedly higher than those obtained from batch and fed-batch cultivation [25]. An exposition on the biosynthetic pathways of some of these bioactive molecules provides a platform for direct manipulation of microalgae for enhanced accumulation of the desired bioactive molecules.

### 2.1. Algae taxonomy

The evolution of algae from primary and multiple secondary endosymbiotic events has probably conferred a unique and spectacular genetic diversity on this group of eukaryotes, which correlates with their phenotypic diversity expressed in size and occupation of diverse ecological niches [26]. Taxonomy [27] has improved tremendously with the emergence of genomic sequencing, which has given rise to bioprospecting for novel bioactive compounds as well as genetic manipulation of relevant species for the economy [26]. The application of genomic data in taxonomy has resolved some difficulties and confusion created by morphologically-dependent taxonomy [28],[29]. For instance, Sequence data generated during a Canadian barcode survey (COI-5P) of the tribes Polysiphonieae and Streblocladieae, a large and taxonomically challenging group of red algae, revealed significant taxonomic confusion and hidden species diversity [30]. According to Bringloe et al. [28], recent multi-locus and genome-scale analyses have revolutionized our understanding of brown algal phylogeny, providing a robust framework to test evolutionary hypotheses and interpret genomic variation across diverse brown algal lineages. Sehgal et al. report [26] that 18S rDNA and internal transcribed spacer (ITS) region of the nuclear genome are two of the most commonly used regions for taxonomically differentiating microalgal species from one another. Despite the trends in algal genome sequencing from 2007 up until 2019, as reviewed by Hanschen et al. [26], and the technological advancement in algal genomic sequencing evidenced by the growing list of publicly available algal genomes, the abundance of genome projects has barely scratched the surface of algal biodiversity [26]. Also, it was observed that the quality of algal genome assemblies is declining [26], which limits the expected taxonomical impacts on the resolution of evolutionary relationships. However, some authors were of the view that integrative taxonomy which involves the use of phylogenetic, morphological, physiological and ecological data was more appropriate for the resolution of taxonomic relationships [29]. By and large, genome sequencing moves that resolve the large gaps in algal biodiversity and evolutionary history will definitely reveal nascent principles of algal biology and evolution, and lead to the discovery of novel proteins, biochemical pathways and untapped natural products [31].

### 2.2. Bioactive molecules in microalgae

Microalgae inhabit complex and extreme environments with varying conditions. They produce various biologically active secondary metabolites and other environment-specific basal metabolites, which other organisms do not produce, to survive these adverse conditions [32],[33], and these substances effectively delay or prevent free radicals' effect on these organisms [34]. Some of these bioactive molecules possess some therapeutic properties such as antioxidant, antibacterial, antiviral, antifungal, antitumor, antimalarial effects and anti-inflammatory properties with great commercialization possibilities in the near future [35]–[37]. According to Gulçin et al. [38], commercial antioxidant supplements like butylated hydroxyl toluene (BHT), butylated hydroxyl anisole (BHA), propyl gallate and α-tocopherol have been applied in oxidative damage mitigation, but Munir et al. [34] suggested that these synthetic antioxidants showed some side effects like carcinogenesis and some damages to the liver. This prompted the need for alternative antioxidants that will protect these organisms from radical reactive oxygen and nitrogen species (OH-, O_2_.-, HO_2_., NO) as well as non-radical forms of oxygen species (H_2_O_2_, and O_2_) with no side effects [39]–[41].

The algal groups are the most genetically diverse group of organisms, and the presence of pigments in these organisms aids them in photosynthetic processes as well as in their grouping. For instance, some contain pigments [42]. The primary pigments in algal groups help them in their growth, thus increasing their biomass, while their ability to produce secondary metabolites protects them from harm during stress conditions. The major stress faced by them is oxidative stress where oxidated radicals are generated in the peroxisomes, chloroplasts or mitochondria as metabolic pathways by-products in these organelles as a result of the high oxidizing activity or intense electron flow which is lethal to their existence. In plants, however, oxidated radicals are also produced in the apoplast and endoplasmic reticulum (ER) besides their cytoplasm. These radicals cause lots of damage to the intracellular molecules such as lipid peroxidation, irreversible protein oxidation and damage in the DNA such as deletion of bases, pyrimidine dimers, cross-links as well as breaks in the DNA strands thereby harming them [43],[44]. Mitigation processes for the removal of the effects of these radicals involve the upregulation of the genes that encode the enzymes superoxide dismutases, peroxidases, reductases and catalases, Filiz et al. [44] further stated.

There is an increase in the current demand for these compounds due to their health benefits when consumed as nutraceuticals or included in functional foods as well as the toxicity of synthetic ones, thus increasing prospecting in algal research for more beneficial biomolecules in microalgae [34]. Both eukaryotic and prokaryotic microalgae such as cyanobacteria have been reported to synthesize numerous secondary metabolites via acetate mevalonic/non-mevalonic acid and shikimic acid pathways [42],[45]. They synthesize some biomolecules like antioxidants when exposed to light and high concentrations of oxygen to mitigate the damaging effects of reactive oxygen species and free radicals, as suggested by Bhosale [46].

## 3. Biosynthesis of bioactive metabolites from microalgae

Several bioactive metabolites are produced by microalgal species at their various growth stages to actively survive the prevailing environmental conditions. For instance, microalgal species such as *Chlorella* sp., *Nannochloropsis* sp., *Spirulina/Arthrospira/Limnospira* sp., *Dunaliella* sp., *Synechococcus* sp., *Phaeodactylum* sp. (Bacillariophyta), *Rhodomonas salina* (Cryptista), *Limnospira maxima* (formerly *Spirulina maxima*) (Cyanobacteria) and *Tetraselmis chuii* (Chlorophyta) produce natural antioxidants with free radical scavenging abilities [12]. Most of these biosynthetic pathways are studied mainly in cyanobacteria and land plants since plants have similar pathways [47]. Some metabolites (such as carbohydrates, proteins and lipids) are produced during their active growth phase through polyketide, mevalonate (MVA)/non-mevalonic or shikimate pathways and subsequently utilized by these organisms during nutrient starvation [37],[46],[48]–[50]. Reports by Huang et al. [51] and Takaichi et al. [52] suggested that carotene and zeaxanthin are present in most microalgal species, and the carotenoid compounds are numerous and serve as biomarkers for their chemotaxonomic classification. The biosynthetic pathways of microalgal carotenoids from phytoene to lycopene are conserved in eukaryotic strains while the downstream synthesis of different carotenoids from lycopene differs according to the carotenoids being produced, as suggested by Tamaki et al. [50].

The five-carbon unit (C5) backbone of carotenoids, Isopentenyl pyrophosphate (IPP), is usually synthesized through two independent pathways, namely, the mevalonate (MVA) pathway or non-mevalonate (1-deoxy-Dxylulose 5-phosphate/2-C-methylerythritol 4-phosphate) (DOXP/MEP) pathway in most microalgae species, except in Euglenophyta species that solely depend on MVA pathway and Chlorophyceae that depends on DOX/MEP pathway to produce carotenoids [52],[53]. According to Takaichi [47] the mevalonate pathway generates IPP from acetyl-coenzyme A, while the non-mevalonate pathway generates IPP by the combination of glyceraldehyde and pyruvate molecules. The IPP then combines with farnesyl pyrophosphate (C15) generated from three IPP molecules to yield geranylgeranyl pyrophosphate (GGPP), a C20 molecule, in a reaction catalyzed by geranylgeranyl pyrophosphate synthase (CrtE or GGPS).

Then, two molecules of GGPP are condensed into phytoene, which is the first carotenoid by phytoene synthase (CrtB or PSY), and this stage is regarded as the rate-limiting step in the carotenoid synthesis, as noted by Rodríguez-Villalón et al. [54]. Furthermore, the enzyme phytoene desaturase (CrtP or PDS) catalyzes the desaturation of phytoene, which is further non-enzymatically photo-isomerized, as suggested by Huang et al. [51]. This molecule is then desaturated by carotene desaturase (CrtQ or ZDS) [50],[51]. The all-trans lycopene can be converted to two carotenoids sub-family carotenes (α and β carotene) and xanthophylls (zeaxanthin, lutein, zeinoxanthin, neoxanthin etc.). Also, Huang and colleagues [51] reported that the formations of α and β-carotenes were catalyzed by the enzyme lycopene α-cyclase and lycopene β-cyclase, respectively. The α and β-carotenes serve as the major precursors in the synthesis of the other carotenoids in the different microalgal species. Although some of the synthetic pathways have been established, some of the enzymes involved in the processes are yet to be identified, especially those carotenoids synthesized from lutein (monadoxanthin, crocoxanthin, siphonaxanthin, monadoxanthin and prasinoxanthin), as suggested by Tamaki et al. [50].

**Figure 1.**
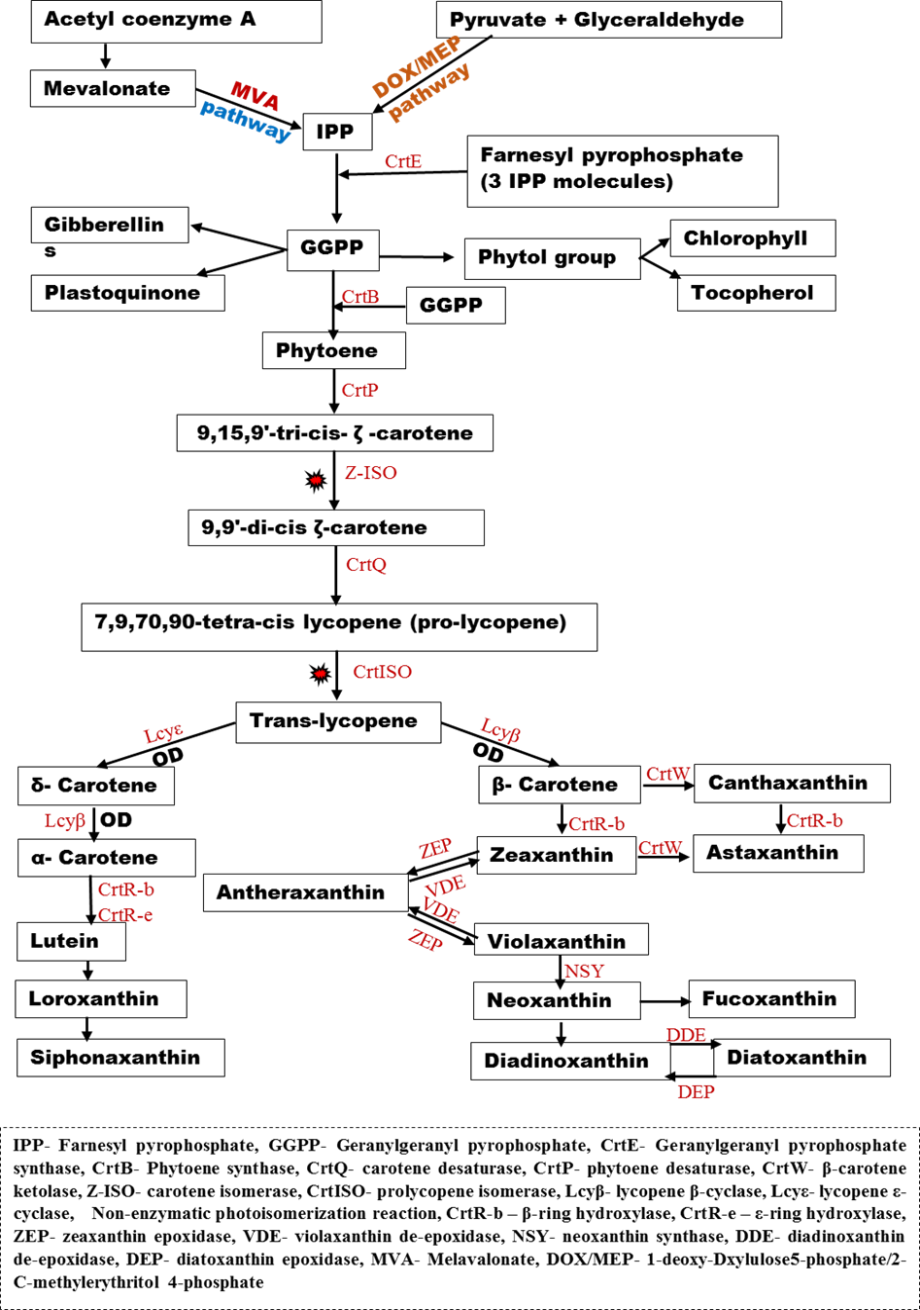
Carotenoids biosynthetic pathway.

**Figure 2.**
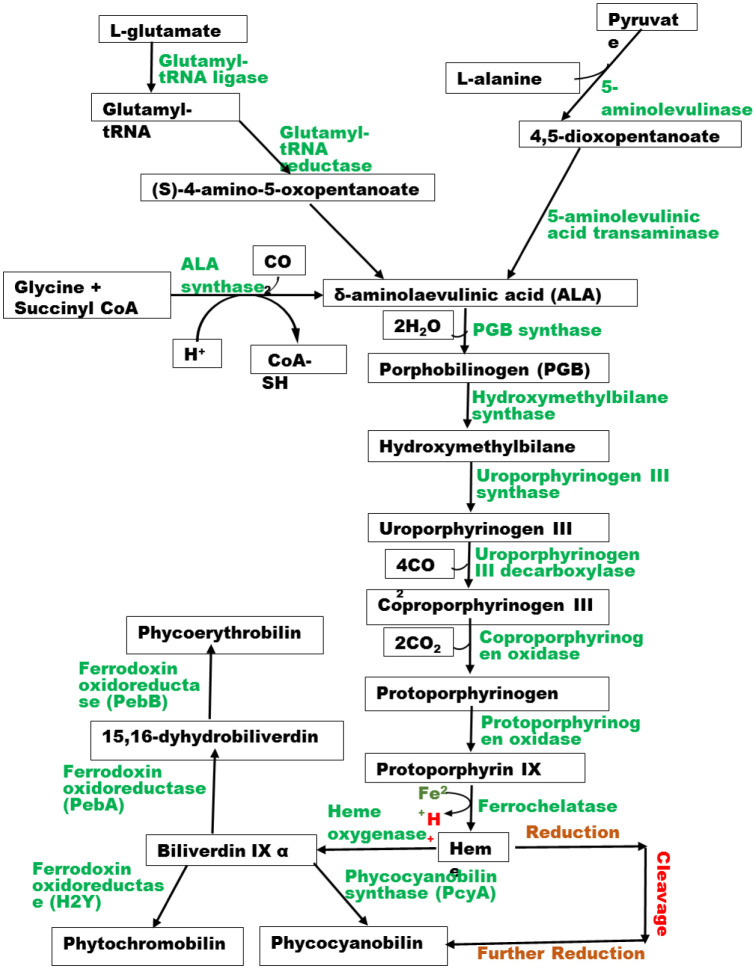
Phycobilin proteins biosynthetic pathway.

The phycobiliproteins synthesis depends majorly on the products of glycolytic pathways such as pyruvate, succinyl coA, L-glutamate and glycine to produce δ-aminolaevulinic acid (ALA) in a series of reactions catalyzed by different enzymes. The heme proteins, according to Li et al. [55], are then metabolized into various phycobilins. Also, antioxidative ascorbate, which is a major electron donor to reactive oxygen species (ROS) scavenging enzyme ascorbate peroxidase (APX), is usually produced in photosynthetic organisms and some animals via D-galacturonate (Euglena) pathway and D-mannose/L-galactose (plant) pathway [50]. These pathways convert D-glucose-6-phosphate into the intermediate L-galactono-1,4-lactone via a series of reaction steps. This intermediate is then dehydrogenated into L-ascorbate by L-galactono-1,4-lactone dehydrogenase, they further stated. The major role played by the ascorbate is to donate electrons that will be used in converting harmful hydrogen peroxide (H_2_O_2_) into water, thus reducing its effect on the system. APX function is similar to that of glutathione peroxidase which accepts electrons from glutathione (GSH) or thioredoxin in H_2_O_2_, lipid peroxide and hydroperoxide detoxification with other GSH-mediated redox regulations [56]. A summary of a developed model of the carotenoid pathway (Figure 1) and phycobilin proteins biosynthetic pathway (Figure 2) based on the aforementioned literature shows the synthesis of various compounds.

## 4. Mechanism of actions of microalgae produced bioactive compounds

The various bioactive compounds produced by microalgae exhibit different modes of action depending on the active ingredient present in them. Microalgae in recent years have been shown to be an important source of functional foods with therapeutic potentials in the treatment of some health disorders and chemoprevention. Most non-communicable diseases that usually develop due to the individual's lifestyle, such as cancer and obesity, can be managed by the application of bioactive molecules of microalgal origin [57]. The presence of antioxidative and anti-inflammatory components makes microalgae a potential agent for the prevention and delay of the onset of some diseases such as cancer and cardiovascular disorders [58],[59]. A wide range of bioactive compounds and their applications are found in Table 1.

**Table 1.** Bioactive compounds from micro-algae and their applications.

S/No	Bioactive Compound(s)	Source(s)	Applications	References
1	Carotenoids: β-carotene Lutein	*Botryococcus braunii, Chlamydocapsa* sp., *Chlorella sorokiniana, Chlorococcum sp., Chondria tenuissima* var*.striolata* (formerly *Chondria striolata*), *Dunaliella* sp, *Scenedesmus* sp., *Muriellopsis* sp., *C. sorokiniana*	Cosmetics additives, natural food coloring agents and health food, Anti-aging, coronary disease prevention, cancer, immune control, retinal and sensory disability enhancement and low-density lipoprotein oxidation inhibition Antioxidant and anti-inflammatory properties	[59],[84]–[86] [87]
	Astaxanthin, Canthaxanthin, Violaxanthin	*Haematococcus* sp., *Chlorolobion braunii* (formerly *Ankistrodesmus braunii*), *Chlamydomonas nivalis, Chlorella vulgaris, Chondria striolata, Monoraphidium* sp., *Tetradesmus obliquus* (formerly *Scenedesmus obliquus*), *Chlamydocapsa* sp., *Chlorococcum* sp., *Neospongiococcum* sp.	Skin protection, eye health enhancement, muscle strength and endurance improvement, protects against oxidative damages, aquaculture feed additives, nutraceuticals, cancer defense, inflammation, metabolic syndrome, diabetes, neurodegenerative and ocular diseases, lung injury, repressed alveolar wall swelling and myeloperoxidase activity. Anti-proliferative activity, Increases Vitamin E, antioxidative, anti-inflammatory and neuroprotective properties	[88]–[92],[5]
2	Phycobiliproteins e.g. Phycocyanin, phycoerythrin, porphyridium and chlorophyll A Proteins (amino acids)	Cyanobacteria, Rhodophyta, Cryptomonads, *Dolichospermum flos-aquae* (formerly *Anabaena. flos-aquae, Caulerpa racemose*, *Ulva lactuca* (formerly *Ulva fasciata*)*, Caulerpa racemosa Spirulina, Porphyridium, Scenedesmus, Chlorella* sp., *Microcystis aeruginosa, Nitzschia incerta, Green algae*	Improves light utilization efficiency, food coloring agents, food antioxidants, humans and plants Functional foods, animal feed supplements, bioplastics production, antioxidant properties, immune activators, prevent atherosclerosis, cancer, and coronary diseases, and also used in photo-ageing protective formulations, cytotoxicity towards tumoral cells	[93]–[112]
3	Vitamins	*Spirulina* sp., *Chlamydomonas* sp., *Chlorella* sp., *Scenedesmus* sp., *Dunaliella tertiolecta*, *Prototheca zopfii* (formerly *Prototheca moriformis*), *T. suecica, Nannochloropsis oculata, Chaetoceros calcitrans*	Antioxidants, Food supplements, Sources of essential vitamins, breast cancer risks reduction, DNA repair and histone methylation as well as chemo-preventive activities, Nutraceuticals, cosmetics	[113],[114]–[117]
4	Polysaccharides e.g., Starch, cellulose, hemicellulose, pectin Sulphated polysaccharides e.g. carrageenan, naviculan, fucoidans, lectin, agar, ulvans, laminaran, galactan, alginate, Stypodiol, taondiol, isoepitaondiol, glycosaminoglycan	*Chlorella vulgaris*, *Fucus vesiculosis* (a marine brown macroalgae), *Margalefidinium polykrikoides* (formerly *Cochlodinium polykrikoides*), *Porphyridium* sp., *Turbinaria conoides, Sargassum wightii*, *Porphyra* sp. (note: *Turbinaria conoides, Sargassum wightii, Porphyra* sp., *Sargassum wightii, Porphyra* sp., are marine macroalgae)	Nanocellulose, biofilters, biofuels, cosmeceuticals, bioplastics, etc. Laminaria sp., Preventive and curative agents for various stages of viral infections such as blockage of reverse transcriptase in HIV infections as well as inhibition of cytopathic effects and cell adhesions during viral infections. Specific algae-derived molecules can be applied in vaccines and antibody production for COVID-19 prevention and cure. Also exhibits antioxidative, immunomodulatory and anti-inflammatory properties	[62],[32],[118]–[121]
5	Phenolic acids e.g. chlorogenic acids, caffeic acids	*Isochrysis* sp., *Chlorella vulgaris, Nannochloropsis* sp.	Anticancer activity inhibits HIV-1 integrase and carcinogenic compounds mutagenicity possesses antioxidant and antispasmodic properties	[122]–[123]
6	Lipids e.g. mono- and polyunsaturated fatty acids (Arachidonic acid, Eicosapentaenoic acid, Docosahexaenoic acid)	*Spirulina, Porphyridium* sp., *Scenedesmus, Lobosphaera incisa* (formerly *Parietochloris incisa*), *Crypthecodinium cohnii, Nannochloropsis* sp., *Schizochytrium* sp., *Ulkenia* sp., *Phaeodactylum tricornutum*	Cardiovascular benefits, mental development and support, anti-inflammatory protects against atherosclerosis, improves the nervous system and brain function, improves infants' growth, functional development and vision	[49],[48],[124],[86]

According to Alam et al. [37], the oceans where microalgae predominantly reside contain a lot of bioactive molecules with preventive, immunostimulatory and immunomodulatory properties for the management of viral infections. Marine algae and microalgae synthesized bioactive molecules such as amino acids and vitamins have also been reported to improve the immune system and thus help in enveloped virus replication inhibition with sulfated polysaccharides and bacterial infections [60],[61]. Carrageenan, the most commonly used polysaccharide in viral infections treatment, and iota-carrageenan have proved to be effective antiviral agents against human immunodeficiency virus (HIV), human rhinovirus (HRV) and human papillomavirus (HPV), acting to prevent these viral agents from initial binding to the host cells during infection stage [37],[61].

The recent COVID-19 global pandemic caused by corona viruses has been reported to be checked by inactivation of the virions before actual viral infection using modified chitosan, especially during low pathogenic infections [62],[63]. Also, chitosan and carrageen were reported to block viral infections by enveloping virions via viral internalization and absorption inhibition, uncoating, improving host immune response [63]. HIV was also reported to be inhibited by sulfated polysaccharides, which also inhibit the activation and expression of receptor pathway epidermal growth factor to suppress coronavirus [64],[65]. This bioactive agent works in various capacities, such as stopping spikes, interaction with receptors in coronavirus infection, as Joseph et al. [66] suggested. The antioxidative properties of these bioactive molecules aid in scavenging free radicals and quenching superoxide radicals generated during oxidative stress conditions [67],[68]. The accumulation of these radicals can cause lipid peroxidation, thus attacking the brain cells due to damage to membranes, polyunsaturated fatty acids (PUFA), and some redox metals like aluminum and iron also generate these radicals by ccatalyzingreactive oxygen species [12]. The damage to PUFA contributes to the progression and pathogenesis of the neurological disorder Alzheimer's disease (AD), associated with neuronal damage, cholinergic dysfunction, oxidative stress, cognitive development protein aggregation and misfolding, neuronal plasticity, synaptic transmission and memory functions, as suggested by Olasehinde et al. [12]. This disease can be treated or managed by the neuroprotective and chemo-preventive potentials of bioactive molecules synthesized by microalgal species.

Microalgal-derived antioxidants, according to Ataie et al. [9], prevent radical-induced neuronal damage and AD progression by scavenging free radicals which harm the brain cells. Also, extracts from Chlorella vulgaris have been reported to be effective in reducing oxidative stress as well as lead-induced oxidative damage in rat brains by decreasing levels of malondialdehyde (MDA) and at the same time increasing glutathione (GSH), catalase (CAT), glutathione peroxidase (GPX) and superoxide dismutase, respectively. These enzymes are important in the mitigation of oxidative radicals' damaging effects in the cells, and their absence makes the cells liable to oxidative damage. The antioxidative properties were linked to their phenolic, carotenoid and fatty acid (myristic, oleic acid and palmitic acids) contents [12],[69],[70]. Peroxidation of these lipids by free radicals generated oxidative stress; metal-induced peroxidation results in the malfunctioning of the brain and other AD-associated symptoms like Aβ-induced neurotoxicity [71],[72]. Also, sulfated polysaccharides prevent cell death and damage to neuronal cells as well as contribute to the antioxidant characteristics of some microalgae. Besides antioxidant ability, anti-inflammatory, immunomodulatory, anticoagulant and anticancer activities have been associated with polysaccharides of microalgal origin [73],[59],[44]. The presence of phytosterols in the cells helps to regulate membrane permeability, integrity and fluidity to carry out their normal physiological processes [74],[12]. As suggested by some authors, sterols exhibit neuroprotective abilities on the nerves, scavenge free radicals and chelate metals, thus inhibiting metal-induced peroxidation in the brain [9],[75],[76].

Memory impairment in AD patients has been reported to be caused by the breakdown of the neurotransmitter acetylcholine by acetylcholine esterase and butyryl choline esterase, which impairs memory function and cognition [77]. Thus, choline esterase inhibitors (such as gallic acids, quercitrin, chlorogenic acids and quercetin) are usually employed in AD management since they stop the activities of esterases and thus improve the availability of acetylcholine in the neurons [31],[75],[78]. The choline esterase inhibitors also stop β-amyloid aggregation, which usually resulted from misfolding of proteins to form plaques, by the activities of transmembrane aspartate protease, β-secretase or β-site APP cleaving enzyme (BACE-1) and γ-secretase or presenilin protein, which affects the neurons [79],[80]. The occurrence of AD with its neurodegenerative effects has been proven to be alleviated by microalgae metabolites with no side effects, as compared to synthetic drugs that have been used until now.

A comprehensive review by Zhou et al. [81] highlighted the several health benefits and mechanisms microalgal compounds use to show a positive effect. They also probed different industries for potential commercial applications. It was concluded that there was compelling evidence for the benefits of microalgal compounds, but further research on their digestive mechanism is required. This has been reiterated by other workers [82]–[83] who posited that fermentation of microalgae has potential, but it is still a fairly new area. It was suggested that more work should be carried out to ascertain the true composition of microalgae to enable manipulation into other products.

## 5. Conclusions

In summary, the biosynthesis of microalgal bioactive molecules aids in their adaptation to different environmental conditions as well as their maintaining various cellular processes for their survival. Also, they have proved to be a source of secondary metabolites with numerous applications, especially in the management of health conditions associated with oxidative stresses, such AD and diabetes. Some species of microalgae have shown the potential of alleviating the impact of the SARS-CoV-2 virus responsible for the global pandemic being experienced in recent years. Although these numerous potentials have been proposed, only a few, such as astaxanthin, extracted from *Haematococcus lacustris* (formerly *Haematococcus pluvialis*) (Chlorophyta), and retinol or b-carotene, from *D. salina* (Chlorophyta), have been applied due to safety and lack of awareness. There is, therefore, the need for the proper education of the populace alongside more complex research to harness these benefits so that they can be effectively applied to the prevention and cure of different diseases.
